# Determination of equi-analgesic doses of inhaled methoxyflurane *versus* intravenous fentanyl using the cold pressor test in volunteers: a randomised, double-blinded, placebo-controlled crossover study

**DOI:** 10.1016/j.bja.2020.12.045

**Published:** 2021-03-04

**Authors:** Harald Lenz, Lars Ø. Høiseth, Marlin Comelon, Tomas Draegni, Leiv A. Rosseland

**Affiliations:** 1 Department of Anaesthesiology, Division of Emergencies and Critical Care, Oslo University Hospital, Oslo, Norway; 2 Department of Research and Development, Division of Emergencies and Critical Care, Oslo University Hospital, Oslo, Norway; 3 Institute of Clinical Medicine, University of Oslo, Oslo, Norway

**Keywords:** acute pain, analgesia, cold pressor test, fentanyl, methoxyflurane

## Abstract

**Background:**

Inhaled methoxyflurane for acute pain relief has demonstrated an analgesic effect superior to placebo. Data comparing methoxyflurane to an opioid are needed. The aim of this study was to determine the equi-analgesic doses of inhaled methoxyflurane *vs* i.v. fentanyl. Both drugs have an onset within minutes and an analgesic effect of 20–30 min.

**Methods:**

Twelve subjects were included in a randomised, double-blinded, placebo-controlled crossover study with four treatments: placebo (NaCl 0.9%), fentanyl 25 μg i.v., fentanyl 50 μg i.v., or inhaled methoxyflurane 3 ml. The subjects reported pain intensity using the verbal numeric rating scale (VNRS) from 0 to 10 during the cold pressor test (CPT). The CPT was performed before (CPT 1), 5 min (CPT 2), and 20 min (CPT 3) after drug administration.

**Results:**

Inhaled methoxyflurane and fentanyl 25 μg reduced VNRS scores significantly compared with placebo at CPT 2 (–1.14 [estimated difference in VNRS between treatment groups with 95% confidence interval {CI}: –1.50 to –0.78]; –1.15 [95% CI: –1.51 to –0.79]; both *P*<0.001) and CPT 3 (–0.60 [95% CI: –0.96 to –0.24]; –0.84 [95% CI: –1.20 to –0.47]; both *P*<0.001). There were no significant differences between the two drugs. Methoxyflurane had significantly higher VNRS scores than fentanyl 50 μg at CPT 2 (0.90 [95% CI: 0.54–1.26]; *P*<0.001) and CPT 3 (0.57 [95% CI: 0.21–0.94]; *P*<0.001).

**Conclusions:**

Inhaled methoxyflurane 3 ml was equi-analgesic to fentanyl 25 μg i.v. at CPT 2. Both resulted in significantly less pain than placebo. Fentanyl 50 μg i.v. demonstrated analgesia superior to methoxyflurane.

**Clinical trial registration:**

NCT03894800

**Editor’s key points**

• Rapid-onset analgesics with short-term effect are useful in the acute setting, for example, trauma and dressing changes, but novel approaches are needed with optimum onset/offset profile and minimal adverse effects.
• An acute pain model (cold pressor test) in volunteers was used to explore the effects of inhaled methoxyflurane, which has been shown to be analgesic compared with placebo.
• In this model, methoxyflurane displayed an analgesic dose–response, similar to fentanyl 25 μg i.v., but less analgesic benefit than fentanyl 50 μg i.v.
• Methoxyflurane had in our study adverse effects like sedation and dizziness, which is typically opioid-related adverse effects (Table 1).

Methoxyflurane (Penthrox®; Mundipharma, Cambridge, UK) is a halogenated ether first used as a volatile inhalational anaesthetic when introduced in the 1960s.^1^ It was withdrawn as an anaesthetic agent because of dose-related nephrotoxicity and hepatotoxicity.^2^ However, methoxyflurane has been used as an analgesic in conscious patients in Australia and New Zealand since the 1970s, predominantly in the emergency department and the pre-hospital setting.^3^^,^^4^ It is licensed in Australia for use in both adults and children, not only for trauma, but also for brief medical procedures.^4^, ^5^, ^6^

Many trauma patients experience pain and insufficient analgesia in the pre-hospital setting,^7^^,^^8^ often because of restricted opioid administration in fear of adverse cardiovascular effects (e.g. hypotension) and respiratory depression. Therefore, it is of interest to investigate other analgesics devoid of these side-effects, such as methoxyflurane.^9^ In many European countries, methoxyflurane has been approved for moderate-to-severe trauma pain.^10^

Methoxyflurane is a clear liquid that is easy to administer after pouring the recommended dose of 3 ml into an inhaler. It has a fast onset of pain relief, less than 5 min, and lasts for approximately 20 min.^11^^,^^12^

In a randomised, double-blinded, placebo-controlled study,^12^^,^^13^ minor trauma patients were randomised to receive methoxyflurane or placebo, both *via* a Penthrox inhaler. In that study, methoxyflurane was well tolerated and superior to placebo with respect to analgesic effect. No serious side-effects (e.g. hypotension or respiratory problems) were observed.

The aim of our study was to determine the equi-analgesic doses of inhaled methoxyflurane compared with i.v. fentanyl. Fentanyl was chosen as a comparator, as it is a widely used drug for acute pain treatment. Both drugs have a fast onset (minutes) and an analgesic effect of approximately the same duration (20–30 min),^11^^,^^12^^,^^14^^,^^15^ making the comparison suitable.

## Methods

The protocol of this randomised, double-blinded, placebo-controlled crossover study was approved by the Regional Committees for Medical Research Ethics in South East Norway (2018/2500; approved on February 8, 2019) and The Norwegian Medicines Agency (reference: 18/18484–7; approved on February 6, 2019), and registered in ClinicalTrials.gov (NCT03894800) and EudraCT (reference: 2018-003939-30). The study was conducted in accordance with the guidelines for Good Clinical Practice,^16^ and was independently monitored by the Clinical Trial Unit at Oslo University Hospital. Data analysis was performed after the final monitor report and the data set was locked.

**Fig 1:**
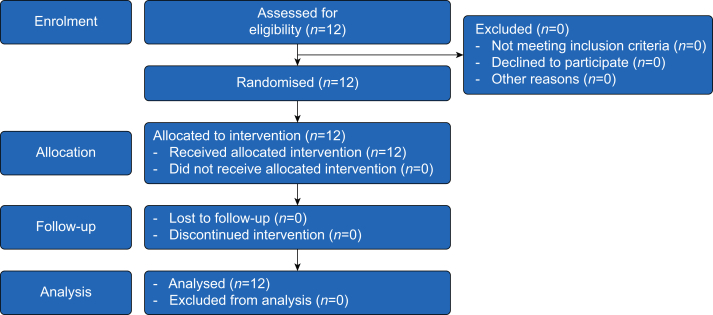
Consolidated Standards of Reporting Trials diagram.

After written informed consent was obtained, 12 healthy volunteers >18 yr were included (Fig 1, Consolidated Standards of Reporting Trials diagram). The participants were recruited through an open invitation to students at the University of Oslo and colleagues at Oslo University Hospital. The subjects' age, sex, weight, height, and medical history were registered. Exclusion criteria were history of liver or kidney disease, chronic illness, use of regular medication, previous substance abuse, use of pain medication and complementary medicine during the past 2 days before a session, participation in other clinical trials the previous 6 months, and known allergies or serious side-effects to opioids or methoxyflurane. The subjects refrained from alcohol 24 h before the sessions.

We performed two pilot studies to determine which doses of fentanyl to compare with methoxyflurane 3 ml. In addition, a retrospective study had compared intranasal fentanyl with inhaled methoxyflurane for visceral pain.^11^ The initial dose of intranasal fentanyl was 0.018 mg compared with inhaled methoxyflurane. Methoxyflurane resulted in the greatest pain score reduction when assessed 5 min after treatment. Intranasal fentanyl has a delay to maximum plasma concentration compared with i.v. fentanyl of 13 *vs* 6 min.^15^^,^^17^ Maximum concentration is also lower after intranasal fentanyl (1.2 *vs* 2.0 ng ml^−1^). Based on the two pilots and these two studies,^11^^,^^15^^,^^17^ we chose to compare methoxyflurane 3 ml with fentanyl 25 and 50 μg i.v.

Before the first treatment, the subjects were familiarised with the cold pressor test (CPT)^18^^,^^19^ and the verbal numeric rating scale (VNRS) for rating pain from 0 to 10 (0=no pain; 10=worst pain imaginable). Side-effects (sedation, dizziness, pruritus, nausea, vomiting, and coughing) were recorded on a verbal rating scale (none=0; some=1; moderate=2; severe=3; very severe=4).

A peripheral venous cannula was placed in the subject's left hand. Pulse oximetry, end-tidal carbon dioxide (ETco_2_), and electrocardiography were continuously monitored. Ventilatory frequency and noninvasive BP were recorded every 5 min.

**Fig 2:**
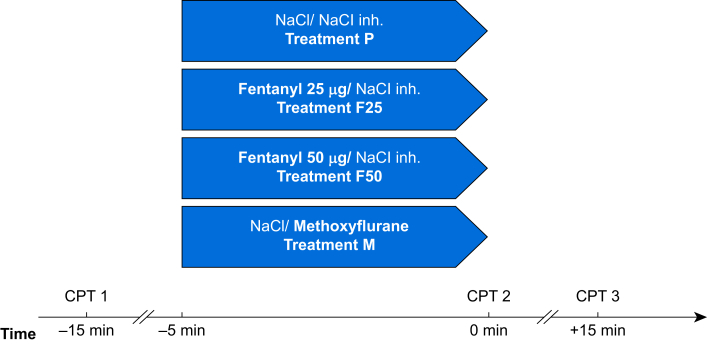
Schematic illustration of the experimental protocol. CPT, cold pressor test; inh., inhalation; NaCl, saline 0.9%; treatment F25, fentanyl 25 μg i.v.; treatment F50, fentanyl 50 μg i.v.; treatment M, inhaled methoxyflurane 3 ml; treatment P, placebo.

The experiment set-up is illustrated in Figure 2. The subjects underwent the four treatments in a randomised order with at least 3 days between each treatment.

Each treatment started with a CPT at time –15 min (CPT 1), followed by 10 min rest. Thereafter (time –5 min), the subjects received either saline 2 ml (NaCl 9 mg ml^−1^; Braun, Hessen, Germany) or fentanyl 25 μg (treatment F25) or 50 μg (treatment F50) (fentanyl; Hameln, Gloucester, UK) i.v. diluted in saline to 2 ml. Concurrently (time –5 min), the subject started inhaling either methoxyflurane 3 ml (treatment M) (Penthrox; Mundipharma) or saline 3 ml for 5 min.

The subjects were instructed to inhale without occluding the dilutor hole for the activated carbon chamber, which could otherwise concentrate the methoxyflurane dose. This is recommended for initial doses in the summary of product characteristics (SPC). According to the SPC, pain relief occurs after six to 10 inhalations. In our model, the subjects inhaled methoxyflurane for 5 min to ensure that 3 ml of methoxyflurane was inhaled, and to ensure that both methoxyflurane and fentanyl reached their maximum clinical effects.^11^^,^^12^^,^^14^^,^^15^ During inhalation of methoxyflurane, saline was administered i.v. In the placebo treatment, subjects received i.v. saline and an inhaler containing saline. When receiving i.v. fentanyl, saline was inhaled. At time 0 min, CPT was repeated (CPT 2), and the third CPT (CPT 3) was performed 15 min thereafter (time +15 min).

In all treatments, the inhalers looked identical, but as methoxyflurane has a characteristic odour, one to two drops of methoxyflurane were applied on the external surface of the primed inhaler before sealing it in a plastic bag. This made the active and placebo treatments indistinguishable to the subjects and the researcher during the sessions.^12^ In addition, before each treatment, the subjects used an oral rinse (SB12; Meda Pharma S. p.A., Monza, Italy) with a strong taste of menthol to hide the characteristic odour of methoxyflurane.

The CPT was conducted using a temperature-controlled bath with circulating 3 °C water (FP 45-HE refrigerated/heating circulator; Julabo Labortechnic, Seelbach, Germany). The subjects submerged their right hand up to the wrist with the fingers abducted for 90 s, and VNRS scores were registered every 10 s.

Randomisation of the treatments was secured by computer-generated codes stored in sequentially numbered envelopes. Block randomisation ensured that each treatment was equally distributed as Treatments 1, 2, 3, and 4 in the 12 subjects. A physician not participating in the handling of the subjects prepared the study drugs in identical opaque envelopes 4 h at the most before use. The Penthrox inhalers were stored in a sealed plastic bag to avoid evaporation. The loss of methoxyflurane from the Penthrox inhalers has been assessed in a previous study, demonstrating a 5% loss after 25 h at 21 °C in a low-density polyethylene bag.^20^

### Statistical analysis

The power analysis was based on a previous study done by our group using CPT for pain assessment.^19^ Our study is a crossover study, and data on standard deviation (sd) of differences between the conditions in a paired comparison were used in the calculation. The primary endpoint was VNRS scores during CPT 2 (5 min after drug administration). The secondary endpoint was VNRS scores during CPT 3 (20 min after drug administration). Other secondary endpoints were side-effects (sedation, dizziness, pruritus, nausea, vomiting, and coughing), *S*po_2_ (oxygen saturation), ETco_2_, MAP, ventilatory frequency, and HR.

The sample size was calculated using the nQuery Advisor® 7.0 (Statistical Solutions, Boston, MA, USA). A sample size of 10 gave 80% power with a 0.05 two-sided significance level to detect a mean VNRS score difference of 0.5 (e.g. treatment means of 6.0 and 5.5), using a paired *t*-test assuming an sd of differences of 0.5. We included 12 subjects to achieve a balanced design with four treatments.

As the aim of the study was to determinate equi-analgesic doses of inhaled methoxyflurane *vs* i.v. fentanyl, the study was powered to detect a mean difference of VNRS score of 0.5. Two studies have demonstrated that clinically significant differences in VAS pain scores are between 0.9 cm (95% confidence interval [CI]: 0.6–1.3) and 1.3 cm (95% CI: 1.0–1.7).^21^^,^^22^ We, therefore, considered the treatments to be equi-analgesic if the difference in VNRS scores was <0.5, as a previous study has demonstrated a lower limit of the CI to be 0.6.^22^

The effects of *treatment* and *CPTnumber* (CPT 1, 2, and 3) on VNRS score were estimated in a non-linear mixed-effects model using the nlme package in R 3.6.3 using RStudio 1.2.5033 (RStudio, Boston, MA, USA).^23^ The VNRS was set to be explained by (α+*treatment* × *CPTnumber*) × (1–exp [*time* × β]), which describes a growth-to-limit function with (α+*treatment* × *CPTnumber*) as the limit, for β<0. We defined this limit, being the VNRS to which the subjects approximated during the CPT, as the outcome. *Treatment* and *CPTnumber* were treated as factors and set as fixed effects using dummy variables with interaction terms. *Time* was the time in seconds into each 90 s CPT with one observation each 10 s on a continuous scale; α and β were set as random effects grouped within subjects. Estimates with CI were calculated using the *glht* function of the multcomp package in R (R Foundation for Statistical Computing, Vienna, Austria).^24^ Comparisons were calculated separately for each treatment and sequence, with *P*-values and CI adjusted for multiple testing by the ‘single-step’ method.^24^ The vital data, with the exception of *S*po_2_, were compared separately for each *CPTnumber* after assigning dummy variables to *treatment* in a linear mixed-effects model. Because of its upper ceiling of 100%, the effect of *treatment* on *S*po_2_ was compared in a Friedman test with *post hoc* pairwise Wilcoxon signed-rank tests with Bonferroni correction. *P*<0.05 was considered statistically significant.

## Results

Recruitment was from April 23, 2019 to October 29, 2019 at Oslo University Hospital. Twelve subjects (six male subjects) were included with age (median [range]) 26 [21–57] yr, height (mean [sd]) 177.2 [8.2] cm, weight (mean [sd]) 72.4 [10.4] kg, and BMI (mean [sd]) 23 [2.2] kg m^−2^.

**Fig 3:**
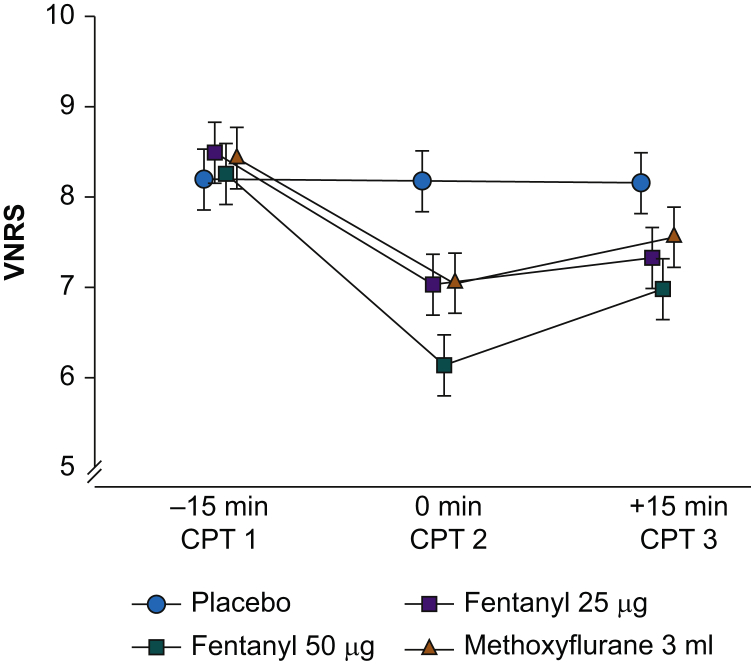
The VNRS scores during the CPTs. The results are presented as estimated VNRS scores in the treatment groups with 95% CI (Supplementary Table 2). CI, confidence interval; CPT, cold pressor test; NaCl, saline 0.9%, VNRS, verbal numeric rating scale.

**Fig 4:**
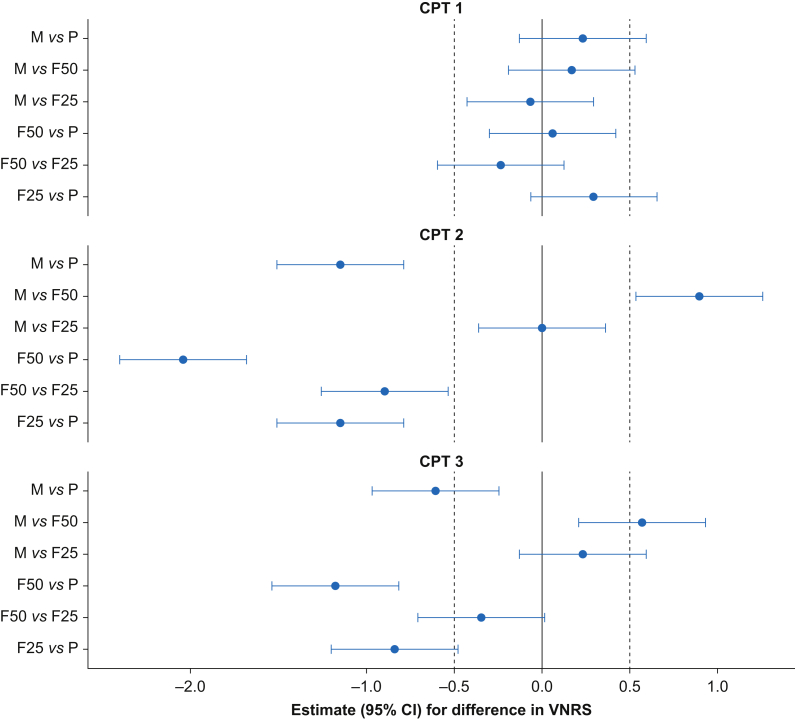
Forest plot of the VNRS scores during the CPTs. Estimates (95% CI) of differences in VNRS scores. Estimates to the left of vertical solid line favour the first treatment group, and *vice versa* (Supplementary Table 2). CI, confidence interval; CPT, cold pressor test; F25, fentanyl 25 μg i.v.; F50, fentanyl 50 μg i.v.; M, methoxyflurane 3 ml; P, placebo; VNRS, verbal numeric rating scale.

The results of the CPTs are presented in Fig 3, Fig 4. Methoxyflurane (M) and fentanyl 25 μg (F25) reduced pain significantly compared with placebo after 5 min (CPT 2) (–1.14 [estimated difference in VNRS scores between the treatment groups with 95% CI: –1.50 to –0.78] and –1.15 [95% CI: –1.51 to –0.79]) and after 20 min (CPT 3) (–0.60 [95% CI: –0.96 to –0.24] and –0.84 [95% CI: –1.20 to –0.47]); all *P*<0.001. M and F25 were not significantly different after 5 min (CPT 2) (0.01 [95% CI: –0.35 to 0.37]; *P*=1.00) and after 20 min (CPT 3) (0.23 [95% CI: –0.13 to 0.59]; *P*=0.34).

M had significantly higher VNRS scores than fentanyl 50 μg (F50) at CPT 2 (0.90 [95% CI: 0.54–1.26]; *P*<0.001) and at CPT 3 (0.57 [95% CI: 0.21–0.94]; *P*<0.001). F50 resulted in significantly less pain compared with F25 at CPT 2 (–0.89 [95% CI: –1.25 to –0.53]; *P*<0.001). There was no significant difference in VNRS scores between F50 and F25 at CPT 3 (–0.34 [95% CI: –0.70 to 0.02]; *P*=0.07).

There were no significant differences in VNRS scores between CPT 1, CPT 2, and CPT 3 in the placebo treatment (CPT 1 *vs* CPT 2; –0.02 [95% CI: –0.35 to 0.31; *P*=0.99], CPT 1 *vs* CPT 3; –0.04 [95% CI: –0.37 to 0.29; *P*=0.95], and CPT 2 *vs* CPT 3; –0.02 [95% CI: –0.35 to 0.30; *P*=0.99]).

**Fig 5:**
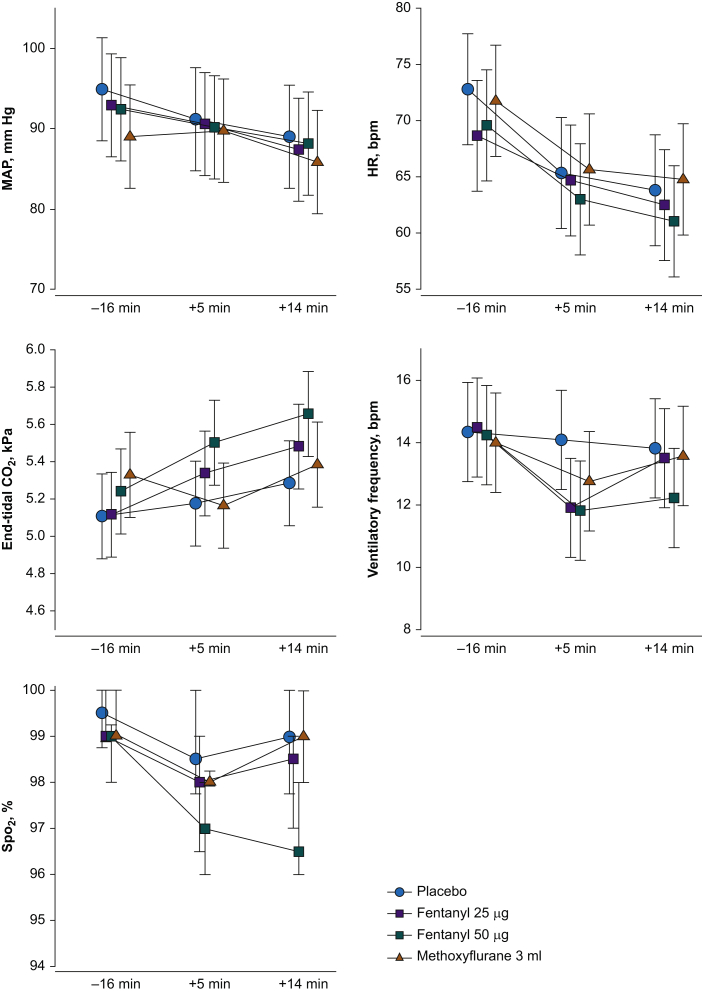
Vital data during the treatments. There were no significant differences in HR, ventilatory frequency, or *S*po_2_ at the time points between the four treatments. There was a significant difference in MAP between methoxyflurane and placebo at time –16 min, *P*=0.02. No other differences were found in MAP. There were significantly lower ETco_2_ in the methoxyflurane treatment compared with fentanyl 50 μg at time +5 min, *P*=0.04, and between placebo and fentanyl 50 μg at time +14 min, *P*=0.02. No other differences were found in ETco_2_. Data for *S*po_2_ are median (25th to 75th percentiles); the other variables are presented as mean (95% CI). *S*po_2_, oxygen saturation. The data are presented with 95% confidence interval.

The side-effects are presented in Table 1. One subject experienced pruritus with treatment F50. Nausea was only registered in the opioid treatments. Most of the subjects experienced sedation and dizziness in the active treatments. In treatment M, four subjects experienced coughing, typically occurring at the beginning of the inhalation period. The vital parameters were compared between the treatments at time –16 min (1 min before CPT 1), +5 min (5 min after CPT 2), and +14 min (1 min before CPT 3). There were no significant differences in HR, ventilatory frequency, or *S*po_2_ between the treatments (Fig 5).

**Table 1:** Side-effects. The table presents the number of subjects who experienced side-effects, classified as none=0, some=1, moderate=2, severe=3, and very severe=4. F25, fentanyl 25 μg; F50, fentanyl 50 μg; M, methoxyflurane 3 ml; P, placebo.

Side-effects	P	F25	F50	M
	0–1–2–3–4	0–1–2–3–4	0–1–2–3–4	0–1–2–3–4
Pruritus	12–0–0–0–0	12–0–0–0–0	11–1–0–0–0	12–0–0–0–0
Nausea	12–0–0–0–0	10–1–1–0–0	10–1–0–1–0	12–0–0–0–0
Vomiting	12–0–0–0–0	12–0–0–0–0	12–0–0–0–0	12–0–0–0–0
Sedation	11–1–0–0–0	3–7–2–0–0	3–4–4–1–0	3–6–3–0–0
Dizziness	12–0–0–0–0	4–6–2–0–0	5–3–4–0–0	4–7–1–0–0
Coughing	12–0–0–0–0	12–0–0–0–0	12–0–0–0–0	8–4–0–0–0

There was a significant difference in MAP between treatments M and P at time –16 min (5.9 mm Hg [95% CI: 0.6–11.0; *P*=0.025]). No other between-treatment differences were found for MAP. There was a significant difference in ETco_2_ between treatments M and F50 at time +5 min (0.33 kPa [95% CI: 0.06–0.66; *P*=0.04]), and between treatments P and F50 at time +14 min (0.38 kPa [95% CI: 0.047–0.70; *P*=0.02]).

## Discussion

Methoxyflurane 3 ml had a superior analgesic effect compared with placebo. As we were unable to demonstrate a statistically significant difference between methoxyflurane and fentanyl 25 μg for the primary endpoint at CPT 2 with the CI being within -0.5 and +0.5, we consider these treatments equi-analgesic (Fig 4). At CPT 3, the CI was outside -0.5 and +0.5, which we assumed in the power analysis. We therefore consider the two treatments as similar.

I.V. fentanyl 50 μg had a superior analgesic effect compared with methoxyflurane 3 ml.

Methoxyflurane has previously demonstrated a superior analgesic effect compared with placebo in a clinical setting.^12^ Furthermore, two recently published studies have demonstrated superior pain relief of methoxyflurane compared with standard analgesic treatment in trauma patients with moderate-to-severe pain.^25^^,^^26^ In the MEDITA (Methoxyflurane in Emergency Department in ITAly) study, the standard treatment was paracetamol 1 g i.v. ketoprofen 100 mg i.v., and the primary outcome was pain assessment at 10 min.^25^ The study has been met with criticism, as the pain assessment was done too early to expect paracetamol and ketoprofen to have taken effect.^27^

A *post hoc* analysis of the MEDITA study has investigated the efficacy of methoxyflurane *vs* i.v. morphine (0.1 mg kg^−1^) for severe trauma pain (VAS score ≥7).^28^ The reduction of pain intensity the first 10 min was superior for methoxyflurane. The measurements were performed 3, 5, and 10 min after randomisation. The morphine dose was administered as an infusion over 10 min, which obviously delayed the clinical effect of morphine. As methoxyflurane was administered immediately after randomisation, the study design clearly favoured the methoxyflurane group.

In many European countries, Penthrox has been approved for the emergency relief of moderate-to-severe trauma pain in conscious adult patients.^10^ We found methoxyflurane 3 ml to be comparable with the low dose of fentanyl 25 μg, indicating that methoxyflurane might be too weak to treat severe pain.

The major strength of our study is the proven assay sensitivity. It is important that single analgesic studies demonstrate both upside and downside sensitivities.^29^ Our study has proved downside sensitivity by finding superior analgesia of methoxyflurane compared with placebo. Furthermore, methoxyflurane was compared with two doses of active comparators: i.v. fentanyl 25 and 50 μg. The finding of superior analgesia for fentanyl 50 μg indicates upside sensitivity. The test assay also found a positive dose–response between the two doses of the active comparators (fentanyl 25 and 50 μg).

Methoxyflurane is not believed to cause hypotension or respiratory depression.^9^^,^^12^ Concerning the dose of fentanyl we found to be equi-analgesic, there were no differences in MAP, ventilatory frequency, *S*po_2_, or ETco_2_ (Fig 5). Fentanyl 25 μg is a low dose of opioid not likely to cause large changes in these parameters.

MAP was significantly lower in treatment M at baseline compared with treatment P, which we believe was a random finding. There was significantly lower ETco_2_ in treatment M compared with treatment F50 at time +5 min, and between treatment P and treatment F50 at time +14 min, indicating that fentanyl 50 μg has a measurable depressive effect on respiration.

Pruritus and nausea were only observed in the opioid treatments. Sedation and dizziness were frequent and similar in treatments M and F25 (Table 1). In treatment F50, more subjects experienced moderate sedation and dizziness than in the other two active treatments. As treatment F50 had superior pain relief compared with the two other active treatments, it is not surprising that there were more side-effects.

Four subjects experienced coughing with methoxyflurane, even though they were instructed to start with two modest inhalations to avoid respiratory irritation. The coughing always occurred at the start of the inhalation. After a brief pause between the inhalations, further coughing was not observed. We believe that this initial respiratory irritation did not affect the total amount of inhaled methoxyflurane.

Methoxyflurane has a characteristic odour, which we tried to hide, but we cannot be sure about whether some of the subjects might have guessed the actual content of the inhaler when inhaling methoxyflurane.

Even though we registered side-effects and vital parameters, one should keep in mind that the study was not powered to demonstrate differences in these parameters.

One important question is the generalisability of our results with respect to acute clinical pain. The external validity of reported pain intensity in experimental pain models is debatable. However, the CPT has been widely used to test opioids, and the model is sensitive to opioid analgesia.^30^ One study presented a statistical approach that successfully demonstrated that findings in experimental pain models can predict clinical analgesia.^31^ To our knowledge, the efficacy of methoxyflurane has never been investigated with the CPT.

In real-life situations, a patient with pain who receives a green whistle (the Penthrox inhaler) might also have the benefits of placebo and distraction, which both have been removed in our study design. A logical next step would be to perform an RCT comparing a single dose of methoxyflurane 3 ml with fentanyl 25 and 50 μg in patients with moderate acute pain after injuries.

In conclusion, inhaled methoxyflurane 3 ml was equi-analgesic to i.v. fentanyl 25 μg 5 min after administration, and we did not find a significant difference between these two treatments 20 min after administration. Both resulted in significantly less pain compared with placebo, and i.v. fentanyl 50 μg resulted in significantly better pain relief compared with methoxyflurane.

## Authors' contributions

Study design: HL, MC, TD, LAR

Recruitment of volunteer participants: HL

Study conduct: HL, TD, LØH, MC

Data collection: HL, TD

Data analysis: LØH, HL

Random code generation: LAR

Paper preparation: all authors

## Funding

Division of Emergencies and Critical Care, Oslo University Hospital, Norway.

## Declarations of interest

The authors declare that they have no conflicts of interest.
