# Supplementary material for: Determination of equi-analgesic doses of inhaled methoxyflurane versus intravenous fentanyl using the cold pressor test in volunteers: a randomised, double-blinded, placebo-controlled crossover study
Source: Br J Anaesth. 2021 Mar 4;126(5):1038–45. doi: 10.1016/j.bja.2020.12.045 (PMC8132882; doi:10.1016/j.bja.2020.12.045)
Supplement: Multimedia component 1 [file mmc1.docx]

| **CPT 1** | F25 | F50 | M |
| --- | --- | --- | --- |
| P | 0.30 (95% CI: -0.06, 0.66; P=0.15) | 0.06 (95% CI: -0.30, 0.42; P=0.97) | 0.23 (95% CI: -0.13, 0.59; P=0.34) |
| F25 |  | -0.23 (95% CI: -0.59, 0.13; P=0.35) | -0.06 (95% CI: -0.42, 0.30; P=0.97) |
| F50 |  |  | 0.17 (95% CI: -0.19, 0.53; P=0.62) |

| **CPT 2** | F25 | F50 | M |
| --- | --- | --- | --- |
| P | -1.15 (95% CI: -1.51, -0.79; P<0.001) | -2.04 (95% CI: -2.40, -1.68; P<0.001) | -1.14 (95% CI: -1.50, -0.78; P<0.001) |
| F25 |  | -0.89 (95% CI: -1.25, -0.53; P<0.001) | 0.01 (95% CI: -0.35, 0.37; P=1.0) |
| F50 |  |  | 0.90 (95% CI: 0.54, 1.26; P<0.001) |

| **CPT 3** | F25 | F50 | M |
| --- | --- | --- | --- |
| P | -0.84 (95% CI: -1.20, -0.47; P<0.001) | -1.18 (95% CI: -1.54, -0.82; P<0.001) | -0.60 (95% CI: -0.96, -0.24; P<0.001) |
| F25 |  | -0.34 (95% CI: -0.70, 0.02; P=0.07) | 0.23 (95% CI: -0.13, 0.59; P=0.34) |
| F50 |  |  | 0.57 (95% CI: 0.21, 0.94; P<0.001) |

Table 2

Statistical results to Figure 3 and 4. The results are presented as estimated differences in VNRS scores between the treatment groups with 95% CI. Should be read as column – row. VNRS = verbal numeric rating scale, CPT = cold pressor test, 95% CI = 95% confidence interval, P = placebo, F25 = fentanyl 25 µg, F50 = fentanyl 50 µg, M = methoxyflurane 3 ml.
